# Phytoextraction and Cd Allocation to the Stem of Woody Species Used in Cacao Agroforestry

**DOI:** 10.3390/plants14071101

**Published:** 2025-04-02

**Authors:** Fabricio E. L. Carvalho, Andrea C. Montenegro, Laura D. Escobar-Pachajoa, Jairo Rojas-Molina, Jorge E. Camacho-Diaz, Gersain A. Rengifo-Estrada

**Affiliations:** 1 Centro de Investigación La Suiza, Corporación Colombiana de Investigación Agropecuaria-AGROSAVIA, Rionegro 687517, Santander, Colombia; ldescobarp@agrosavia.co (L.D.E.-P.); jrojas@agrosavia.co (J.R.-M.); jecamacho@agrosavia.co (J.E.C.-D.); grengifo@agrosavia.co (G.A.R.-E.); 2 Centro de Investigación Tibaitatá, Corporación Colombiana de Investigación Agropecuaria-AGROSAVIA, Mosquera 250047, Cundinamarca, Colombia; amontenegro@agrosavia.co

**Keywords:** cadmium, *Cariniana pyriformis*, *Cedrela odorata*, heavy metal, phytoremediation, SAF, *Swietenia macrophylla*, *Terminalia superba*, *Theobroma cacao*

## Abstract

Global cacao production, primarily led by African countries, is facing a crisis, which presents growth potential for South American countries like Colombia, Peru, and Ecuador. However, a significant challenge for these countries is cadmium (Cd) contamination in cacao beans. Agroforestry systems with cacao (CAFSs) improve soil health and can remediate Cd through tree phytoextraction. Effective phytoremediation requires Cd-tolerant, high-biomass species and preferential Cd allocation to stems. This study evaluated the phytoremediation potential of four forest species (*Cariniana pyriformis* Miers, *Terminalia superba* Engl. and Diels, *Swietenia macrophylla* King, and *Cedrela odorata* L.) under cadmium (Cd) exposure. *C. pyriformis* exhibited hypertolerance, showing minimal biomass reduction (less than 15%, changing from 1.619 to 1.343 g plant^−1^) under excess Cd conditions, compared to *Cedrela odorata* and *T. superba*, which showed significant biomass reductions. *C. pyriformis* and *T. superba* showed notable Cd accumulation in stems (652.99 and 635.39 mg Cd kg^−1^), an essential feature for wood tree-mediated phytoextraction, while *C. odorata* allocated more Cd to leaves (35.35 mg Cd kg^−1^). *C. pyriformis* maintained high photosynthesis (12.8 μmol CO_2_ m^−2^ s^−1^), light use efficiency (0.086 mol CO_2_ mol photons^−1^), and an increased relative growth rate (0.575 g g^−1^ day^−1^) under Cd exposure. Overall, *C. pyriformis* demonstrated significant potential for use in phytoremediation due to its high Cd tolerance (84%), efficient allocation to stems (17%), and sustained physiological performance under Cd exposure. Conversely, *C. odorata* allocates Cd to leaves (16%), which can reintroduce Cd into the soil, and exhibits a low tolerance index (54%) under higher cadmium contamination. Further studies are still needed to understand the specific mechanisms of Cd accumulation in stems of promising species like *C. pyriformis* and *T. superba*.

## 1. Introduction

Cadmium (Cd) is an unessential trace element in plants, considered one of the most toxic species and poses a significant risk to the environment due to its high mobility. This element is distributed in soils worldwide [1]. In cacao soils from south America, Cd contamination has been attributed to geogenic or anthropogenic sources [1]. Generally, the origin of this cadmium is believed to be geogenic due to the low use of fertilizers in many Latin American countries [1], but the degree of Cd contamination from human actions such as the use of contaminated fertilizers is currently unknown. Many studies have documented the translocation of Cd from soil, especially under low pH conditions, to plant shoots via roots and its accumulation in edible plant organs [2,3,4,5]. In fact, some studies suggest that Cd loading into cacao may occur via direct xylem uptake; however, the pathways through which this metal is taken up and loaded into cacao is not fully understood to date. More recently, studies using Cd isotopes revealed that Cd in cacao possibly originates from a redistribution via the phloem, transported from the stem, branches or mature leaves and not directly from roots [2]. Regardless of the pathways Cd accumulates in cacao, this heavy metal is probably carcinogenic and harmful to human health [6].

Cacao beans are one of the most essential exported agricultural commodities produced by farmers from several countries in South America, mainly Brazil, Colombia, Ecuador, Peru, and Venezuela [4]. For some years now, concern has grown about the presence of Cd in beans and consequently in the products derived from their processing due to the limits established by the European Food Safety Agency (EPSA), especially from the cacao-growing countries of Central and South America [7]. In Colombia, cacao cultivation is performed under an agroforestry system (AFS) [8] and is worked on by small farmers with an average of 3.5 hectares, where these systems have been recognized as an option for improving the livelihoods of rural families [9,10].

Regarding the economic value for farmers, in Africa, Indonesia, and Latin America, cacao-AFSs have been used as alternatives to improve the well-being of the local population by adding commercial value through multiple-use species such as fruit trees (oranges, avocados, dragon fruit, black pepper) and those for timber purposes, e.g., cedar and mahogany [11,12]. Each system’s botanical composition and structural diversity of cacao-AFSs are unique, although some vegetation patterns and structural types can be differentiated [10,13]. In Central America, it is suggested that AFSs play a vital role in conserving tree diversity and highlights the importance of crop management, species selection, and farmer participation in conservation efforts [14].

As Asitoakor et al. [15] highlighted, selecting shade species in agroforestry systems with cacao is essential to maintaining high yields in systems with low inputs. In Ghana, they found that *Cedrela odorata*, *Terminalia superba*, *Khaya ivorensis,* and *Milicia excelsa* increased yield when used in CAFSs compared to a full-sun cacao cultivation system. González-Valdivia et al. [16] found that the most common species recorded in CAFS studies with cacao in Central America are *Cedrela odorata* (eight countries), *Bursera simarouba,* and *Cordia alliodora* (both in seven countries), followed by *Tabebuia rosea* (in six countries), *Enterolobium cyclocarpon,* and *Guazuma ulmifolia* (in five countries each). Additionally, *Cariniana pyriformis* Miers, commonly known as Colombian mahogany or Abarco, is a tree species from the *Lecythidaceae* family. The intense exploitation of its wood for timber in Colombia, Panama, and Venezuela has led to significant habitat loss for this species. In Colombia, this species is classified as critically endangered. Since 2010, efforts to promote its conservation have included agroforestry systems with cacao and the establishment of pure plantations [17].

AFS cacao plants have the property of improving soil health by providing significant amounts of leaf litter, which contributes to the formation of soil organic matter, in addition to promoting sustainability in tropical agroecosystems [18,19]. In these systems, the presence of other species can affect the absorption of available elements from the soil through competition for light, water, nutrients, and contaminants. Trees can then act as natural cadmium phytoremediators. In the literature, different examples illustrate the remedial potential of trees in soils contaminated with heavy metals. Some plant species can tolerate the presence of high concentrations of heavy metals, such as cadmium, in the soil. This adaptation allows the plant to extract, translocate, and compartmentalize large concentrations of toxic elements such as cadmium in its tissues or reduce the cadmium available for the main crop such as cacao [20]. However, only 0.2% of angiosperms are considered hyperaccumulators of heavy metals, meaning that prospecting forestalling species adequate to CAFSs and phytoextractors is not easy [21].

Trees are ideal plants for a long-term remediation strategy. Some research has shown that certain high-biomass species tolerate However, only 0.2% of angiosperms are considered hyperaccumulators of heavy metals, making it challenging to identify species that are adequate for CAFSs and phytoextractors at the same time. In this context, tree species such as *Salix viminalis*, *Populus* sp., and *Swietenia macrophylla* are promising for implementing phytoremediation techniques [22,23]. Using heavy metal-accumulating plants is a possible strategy for decontaminating soils [24,25]. These plants have an extensive root system that develops in different soil strata, potentially guaranteeing more efficient extraction. Likewise, some species considered phytoextractors can interact, in a mutualistic relationship, with microorganisms, nitrogen-fixing bacteria, and arbuscular mycorrhizal fungi, which facilitate their adaptation to highly contaminated soils [20]. Moreover, working in Bolivia, Gramlich et al. [26] found that cacao trees under agroforestry systems accumulated less Cd in the leaves than under monocultures, which can be attributed to the higher competition with other trees for Cd uptake in the agroforestry systems as compared to the monocultures. Although the cadmium content in leaves is not necessarily linearly correlated with the content in cacao beans [27], the associated shade species in CAFSs can potentially reduce Cd levels in cacao through phytoextraction. Indeed, phytoremediation of Cd-contaminated soil using Cd hyperaccumulators is a practical, low-cost, and sustainable method to prevent threats to ecological safety, agricultural production, and human health from Cd toxicity [5]. However, for effective phytoremediation, the selected plants should ideally tolerate and accumulate high levels of Cd while maintaining rapid growth rates to translocate the excessive Cd from the soil into easily harvestable aerial parts [28].

In previous work, our group evaluated the potential for hypertolerance, phytoextraction, and allocation of cadmium under nutrient solution conditions in four forest species of regional relevance for agroforestry systems with cacao: *Tabebuia rosea*, *Terminalia superba*, *Albizia guachapele*, and *Cariniana pyriformis* [29]. The data obtained in the study revealed that *T. superba*, *A. guachapele*, and *C. pyriformis* are moderately tolerant to cadmium (TI > 0.6), and *T. rosea* is a sensitive species (TI < 0.35). Moreover, considering all the favourable features exhibited by *T. superba*, such as Cd hyperaccumulation, high tolerance index, low Cd concentration in leaves, and high Cd allocation to the stem (harvestable as wood), this species was considered to have a high potential for cadmium phytoextraction in cacao agroforestry systems [29]. Nevertheless, considering that increases in cadmium concentration in plants can also be derived from a reduction in biomass due to the occurrence of stress, it is important to consider hyperaccumulation and hypertolerance as important complementary characteristics in the evaluation of potential phytoextractor species [29].

In the present study, a more detailed approach was carried out on the physiological response of four CASF species under cadmium-contaminated substrate conditions: *C. pyriformis* and *T. superba*, due to the phytoextraction potential previously highlighted in hydroponic conditions [29]; *C. odorata*, due to its high occurrence as a forest species in Colombian CAFSs [15,16]; and *S. macrophylla* as a reference, due to its phytoextraction potential previously reported in the literature [22]. The main objective of this study was to explore the potential for cadmium accumulation in regionally important forest species for cultivation in agroforestry with cacao designs. This was performed with the aim of seeking potential nature-based solutions to the problem of excess cadmium in cacao systems. The data obtained here, including tolerance, translocation, and allocation of cadmium in the stem, physiological changes in stomatal dynamics and photosynthetic activity, and growth, corroborate a potential role of phytoextraction for *T. superba* and *C. pyriformis*, essential forestry species in the context of agroforestry systems with cacao.

## 2. Results

### 2.1. Cariniana pyriformis Exhibited Hypertolerance to Excess Cadmium

The present study sought to evaluate three crucial markers for the selection of forest species that may play a phytoremediation role in CAFSs: (1) potential for hypertolerance to substrate contaminated with CdCl_2_; (2) potential for Cd hyperaccumulation and preferential allocation in stems; and (3) physiological resilience under CdCl_2_ exposure. For this, an artificial substrate contamination gradient was created, which consisted of three treatments: (a) reference with 0.41 mg Cd kg^−1^ soil; (b) “T1” reaching 7.6 mg Cd kg^−1^ soil; and (c) “T2”, which presented 17.4 mg Cd kg^−1^ soil (Figure S1). These values were selected based on averages and maximums quantified in soils from different studies reported in the literature for cacao crops.

**Figure 1:**
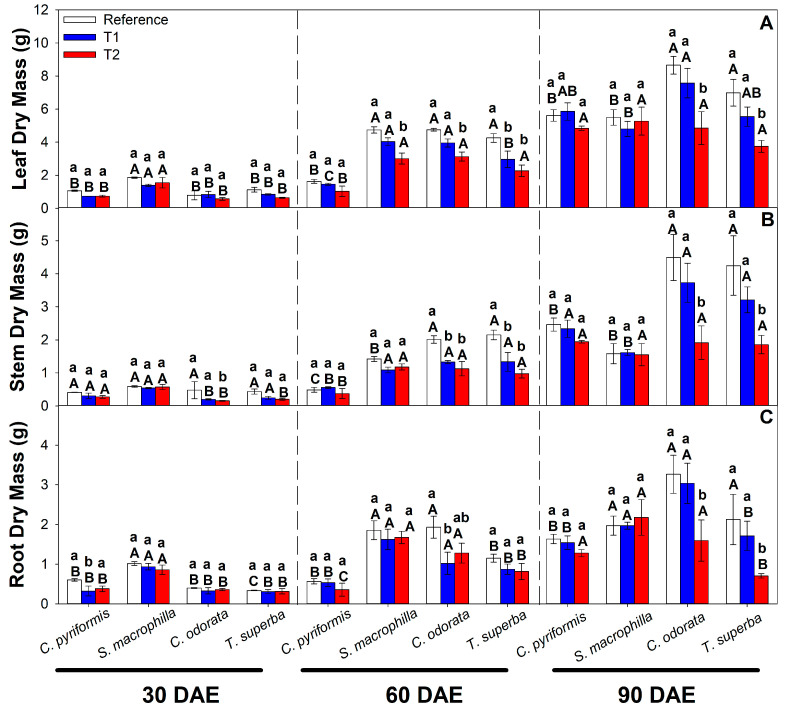
Dry mass in leaves (**A**), stems (**B**), and roots (**C**) of four forest species (*C. pyriformis*, *S. macrophylla*, *C. odorata*, and *T. superba*) exposed to contrasting levels of cadmium at 30, 60, and 90 days after cadmium exposure. The bars represent mean values ± standard error (*n* = 3). Different capital letters mean significant differences between plant species at the same cadmium contamination level, and different lowercase letters represent significant differences between cadmium treatments in the same plant species, according to Tukey’s test (*p* < 0.05).

To estimate the potential tolerance of the species, the dry mass of leaves, stems, and roots was quantified at 30, 60, and 90 days after contamination with CdCl_2_. Overall, a trend where increasing cadmium levels result in lower biomass was observed, but this is not uniform across all the species evaluated here (Figure 1). In terms of leaf dry mass, *C. odorata* and *T. superba* were the species that showed the most significant reduction after 90 DAEs of cadmium exposure (T2), approximately 40% and 50%, changing from 1.443 to 0.808 g and from 1.165 to 0.624 g, respectively, as compared with reference treatment (Figure 1A). *C. odorata* and *T. superba* also showed a substantial reduction in dry mass of stem, 60% and 40%, changing from 0.748 to 0.319 g and from 0.707 to 0.309 g, respectively (Figure 1B). In roots, these species also exhibited a decrease in dry mass of approximately 50% each one, changing from 0.544 to 0.266 g and from 0.354 to 0.117 g, respectively, after 90 days of high cadmium (T2) exposure and compared to the reference treatment (Figure 1C). In contrast, *C. pyriformis* only showed a slight reduction (less than 15%) in the dry mass of leaves, stems, and roots after 90 days of exposure to excess cadmium (T2) compared to the reference treatment, changing from 0.936, 0.411, and 0.272 g to 0.807, 0.323, and 0.213, respectively. *S. macrophylla* did not show significant differences in biomass at T1 and T2 compared to the reference treatment (Figure 1) after 90 days of exposure to cadmium.

The changes in stem diameter, number of leaves (or leaflets), and average leaf area, in turn, showed few significant differences between the different contamination levels with excess cadmium (Table S1). Overall, the data do not show significant differences in these variables in response to the cadmium treatments for the four species evaluated up to 60 days after exposure. However, after 90 DAEs, *T. superba* shows a significant drop in diameter, from 4.4 in reference to 3.2 mm in T2, a decrease of approximately 25%. *C. pyriformis*, in turn, showed a significant reduction in leaf area, from 17.1 cm^2^ in the reference treatment to 9.8 cm^2^ in the T2 treatment, evidencing a decrease of approximately 40%. On the other hand, *S. macrophylla* and *C. odorata* did not exhibit significant changes in stem diameter, number of leaves (or leaflets), and average leaf area in response to cadmium exposure (Table S1).

Regarding the tolerance index (TI), all four forest species presented values above 80% after 90 days of exposure to T1 (7.6 mg Cd kg^−1^ soil). This implies a relatively high tolerance to cadmium in all species evaluated, since the contamination values associated with T1 are relatively high. Nevertheless, when exposed to T2 (17.4 mg Cd kg^−1^ soil), some species present a reduction in the values associated with the tolerance index. *S. macrophylla* and *C. pyriformis* presented the best results regarding the tolerance index, presenting TI equivalent to 98 and 84% in T2, after 90 days of exposure. On the other hand, *C. odorata* and *T. superba* were the species that reduced TI the most, presenting 54% and 52%, respectively, in T2 after 90 days of cadmium exposure (Table 1).

Comparing the growth of different species can be challenging due to genetically based differences. Therefore, the relative growth rate was determined to estimate the more efficient species in biomass production under cadmium exposure. The results revealed that *C. pyriformis* presented a phase of slow growth that coincided between 30 and 60 days after exposure to cadmium (T1 and T2), but that was not related to the presence of this metal, as it was also present in the reference treatment (Table S2). During the 60- to 90-day interval, only *C. odorata* and *T. superba* showed a reduction in RGR in the presence of excess cadmium (T2). Under such conditions, *C. odorata* exhibited a decrease in RGR from 0.0209 in reference to 0.0119 in T2, and *T. superba* decreased from 0.0181 to 0.0152 (Table S2). *C. pyriformis*, on the other hand, presented RGR values in the 60–90 interval up to 34% higher in T2 compared to the reference, changing from 0.0430 to 0.0575 (Table S2). These RGR data, combined with all the analyzed growth data, suggest that *C. pyriformis* and *S. macrophylla* are the most tolerant species among those evaluated.

### 2.2. Cariniana pyriformis and Terminalia superba Showed Hyperaccumulation and Marked Allocation of Cadmium to the Stem

Regarding the cadmium content in leaves, stems, and roots, the four forest species showed an increasing trend depending on the increased level of cadmium in the substrate and the time of exposure to these conditions (Table 2). In T2, after 90 days of exposure, the cadmium content in leaves was highest in *C. odorata* (35.3 mg Cd kg^−1^), followed by *T. superba* (13.3 mg Cd kg^−1^), *S. macrophylla* (6.7 mg Cd kg^−1^), and *C. pyriformis* (5.5 mg Cd kg^−1^). On the other hand, in stems, the species that exhibited the highest content was *C. pyriformis* (93.7 mg Cd kg^−1^), followed by *T. superba* (78.8 mg Cd kg^−1^), *C. odorata* (44.9 mg Cd kg^−1^), and *S. macrophylla* (28.8 mg Cd kg^−1^) (Table 2). This same order of species was also observed in terms of cadmium content in roots, where *C. pyriformis* exhibited (653 mg Cd kg^−1^), followed by *T. superba* (635 mg Cd kg^−1^), *C. odorata* (613 mg Cd kg^−1^), and *S. macrophylla* (295 mg Cd kg^−1^).

**Figure 2:**
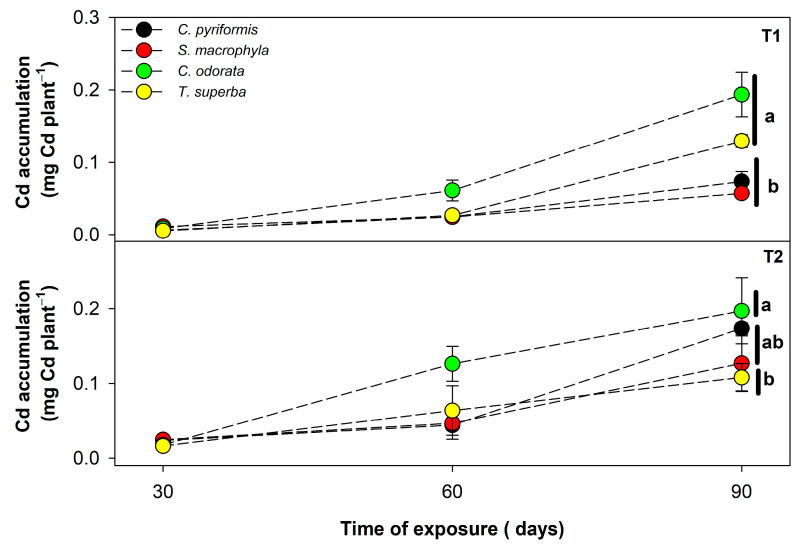
Cadmium total accumulation in four forest species (*C. pyriformis*, *S. macrophylla*, *C. odorata*, and *T. superba*) exposed to contrasting levels of cadmium contamination (T1 and T2) for up to 90 days. Data are presented based on the Cd content determined (mg·kg^−1^) multiplied by the accumulated dry weight (g) per plant at 30, 60, and 90 days after cadmium exposure. Results are expressed in mg cadmium per plant. The circles represent mean values ± standard error (*n* = 3). Different lowercase letters mean significant differences between plant species at 90 days after cadmium exposure, according to Tukey’s test (*p* < 0.05).

Considering that the phytoextraction potential of a plant can be defined both by the cadmium content that it can support in tissues and can also be affected by the growth rate of the plants, the total absolute cadmium accumulated per unit of plant in each forest species was also estimated and evaluated (Figure 2). In general, the four species tended to accumulate cadmium over time, regardless of the level of contamination by CdCl_2_. In T1 (7.6 mg Cd kg^−1^), however, *C. odorata* and *T. superba* were the species that managed to accumulate more cadmium in a plant unit, where *C. odorata*, followed by *T. superba* may have accumulated between 2 and 4 times more cadmium total per plant (0.19 and 0.13 mg Cd plant^−1^, respectively) as compared to *C. pyriformis* (0.07 mg Cd plant^−1^) and *S. macrophylla* (0.06 mg Cd plant^−1^)—(Figure 2). On the other hand, in T2 (17.4 mg Cd kg^−1^), where substrate Cd levels were much higher, there was less difference between the absolute amount of cadmium accumulated by the different species evaluated, but *C. odorata* exhibited a significant difference in Cd accumulation (0.20 mg Cd plant^−1^) as compared to *T. superba* (0.11 mg Cd plant^−1^)—(Figure 2).

**Figure 3:**
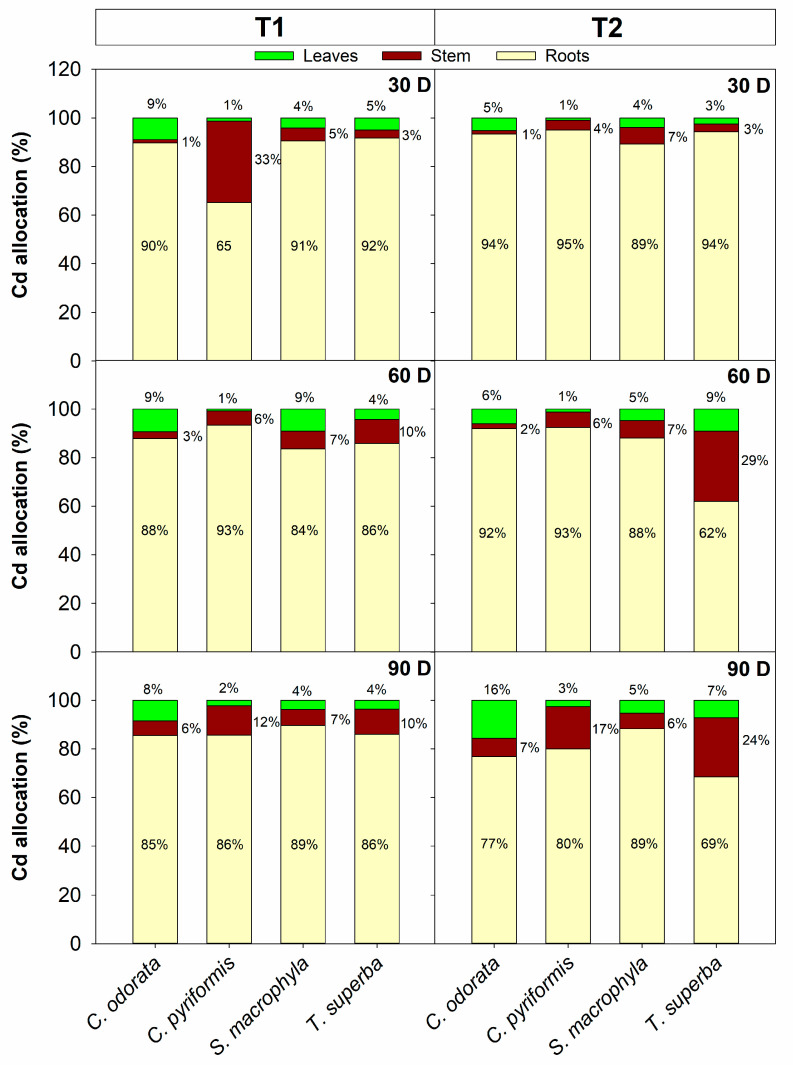
Cadmium allocation in leaves, stems, and roots of four forest species (*C. pyriformis*, *S. macrophylla*, *C. odorata*, and *T. superba*) exposed to contrasting levels of cadmium contamination (T1 and T2) for up to 90 days. Data are presented based on the Cd content determined (mg·kg^−1^) multiplied by the accumulated dry weight (g) in each plant organ (leaves, stem, and roots) at 30, 60, and 90 days after cadmium exposure. Results are expressed in mg cadmium per plant. The bars represent mean values ± standard error (*n* = 3).

In addition to accumulating cadmium, forest species that may be interesting for phytoextraction strategies associated with SAF with cacao must minimize the allocation of this metal to the leaves, prioritizing harvestable tissues, in this case, the stem wood. In T1, after 90 days of exposure, *C. pyriformis* and *T. superba* were the species that allocated the most cadmium to stem tissues, allocating 12% and 10%, respectively, of the total absolute cadmium accumulated per plant (Figure 3). On the other hand, under these same contamination conditions, *C. odorata* was the species that allocated the most cadmium to the leaves, reaching 8% of the absolute total accumulated per plant. In T2, also after 90 days of exposure, the species that allocated the most cadmium to the stem were *T. superba* (24%) and *C. pyriformis* (17%). Also, in T2, the species that allocated the most metal to leaves was *C. odorata* (16%) (Figure 3). The bioconcentration factor (BCF) of cadmium in leaves was also estimated, which is a relationship between the cadmium content in leaves and that of the soil. *C. odorata* was the species that presented a much higher leaf BCF compared to the other species at both levels of soil contamination (T1 and T2), reaching approximately 5 to 3 times higher when compared to the values observed in *C. pyriformis* and *S. macrophylla* (Table S3), corroborating the high allocation of cadmium in photosynthetically active tissues by *C. odorata*.

Finally, in the relationship between Cd in soils and the concentration of Cd in the different tissues, as expected at the beginning of sampling, a significant correlation (*p* < 0.05) was found between the accumulation of Cd in roots (r = 0.89) and the soil (Table S4). Furthermore, in the second sampling, a significant correlation also appears (*p* < 0.05) between the accumulation of Cd in stems (r = 0.91) (Table S4). However, the correlation remains with the root content (r = 0.86) until the final sampling (r = 0.91, *p* < 0.05), which is desirable because, in this experiment, a substrate contaminated with cadmium was used, which is available around the root. These results perfectly align with what has been reported in the literature [30,31], further reinforcing their credibility.

### 2.3. Cariniana pyriformis Showed High Photosynthetic Resilience Against Cadmium Toxicity but a Greater Susceptibility Related to Leaf Respiration

To deepen the understanding of the physiological mechanisms affected by the exposure of these four forest species to excess cadmium in the substrate, photosynthetic response curves to light (A-PPFD, gSw-PPFD, and WUE-PPFD) were performed after 30 (Figure S2), 60 (Figure S3), and 90 (Figure S4) days after exposure to T1 and T2. From the modelled A-PPFD curves [32], the values of maximum light-limited photosynthesis rate (Amax), maximum light use efficiency (LUE), and leaf mitochondrial respiration rates in darkness (Rdark) were estimated.

**Figure 4:**
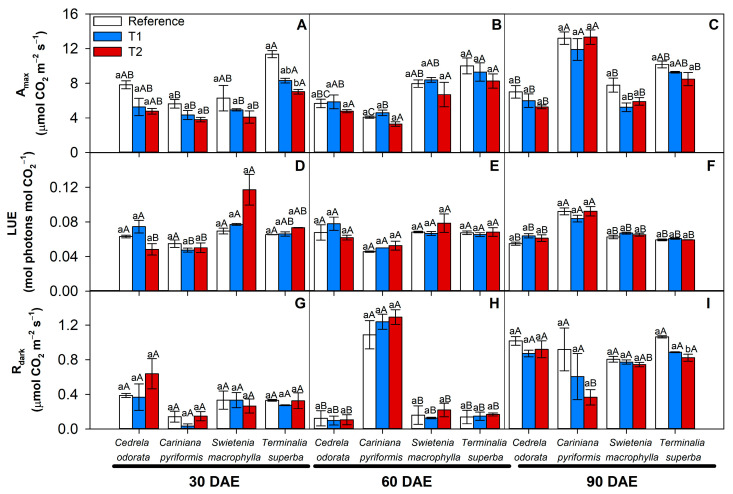
Photosynthetic parameters in four forest species (*C. pyriformis*, *S. macrophylla*, *C. odorata*, and *T. superba*) exposed to contrasting levels and exposure time of cadmium. Maximum light-limited photosynthetic rate, Amax (**A**–**C**); light use efficiency, LUE (**D**–**F**); and dark mitochondrial respiration, Rdark (**G**–**I**). The bars represent mean values ± standard error (*n* = 3). Different capital letters mean significant differences between plant species at the same cadmium contamination level, and different lowercase letters represent significant differences between cadmium treatments in the same plant species, according to Tukey’s test (*p* < 0.05).

Cadmium treatments induced a lower Amax trend in the four species tested here during the first 60 days of exposure (Figure 4A,B). After 90 days after exposure, however, despite this trend, no significant difference was detected regarding the effect of cadmium on Amax rates in any of the forest species (Figure 4C). Additionally, no significant differences were recorded in terms of LUE in response to treatment with excess cadmium in any of the shade species (Figure 4D–F). Among the different species, however, *C. pyriformis* stood out because it presented a higher Amax (12.8 μmol CO_2_ m^−2^ s^−1^) and light use efficiency (LUE) (0.086 mol CO_2_ mol photons^−1^) as compared to *T. superba* (9.3 and 0.06, respectively), *S. macrophylla* (6.3 and 0.065, respectively), and *C. odorata* (6.1 and 0.060, respectively) after 90 days, despite the cadmium contamination (Figure 4C,F).

In addition, no significant differences in Rdark were reported in the presence of cadmium treatments (Figure 4G,H). However, at 60 days, a substantial increase in Rdark was observed in *C. pyriformis*, which presented values around six times higher (1.2 μmol CO_2_ m^−2^ s^−1^) than the other species at that same sampling point (approximately 0.15 μmol CO_2_ m^−2^ s^−1^) regardless of the level of cadmium contamination (Figure 4H). In parallel, *C. pyriformis* was the only one to show a 60% reduction in Rdark (0.92 μmol CO_2_ m^−2^ s^−1^), induced by T2 after 90 DAEs, as compared to the reference treatment (0.23 μmol CO_2_ m^−2^ s^−1^) (Figure 4I). The changes in these photosynthetic parameters could be associated with changes in stomatal aperture and, consequently, water use efficiency (Figure S4).

Regarding the light requirements identified for the four species, the saturation threshold constant (IK) varied from 173.5 (µmol photons m^−2^ s^−1^) in *T. superba* to 125.6 in *C. pyriformis*, 116.2 in *C. odorata*, and finally 82 in *S. macrophylla* (Table S5). The light required for 90% of maximum photosynthesis (L90) according to A-PPFD, in turn, was approximately 1650 (µmol photons m^−2^ s^−1^) in *T. superba*, followed by 1280 in *C. pyriformis*, 1080 in *C. odorata*, and finally 790 in *S. macrophylla*. *T. superba* showed reductions in IK and L90 but only in T2 at the showing point of 30 days of exposure to cadmium. After 90 days of Cd exposure, only *S. macrophylla* significantly reduced IK and L90 compared to the reference treatment (Table S5).

### 2.4. Correlation Analysis Revealed a Strong Negative Correlation Between Photosynthetic Variables and the Percentage of Cadmium Allocation in Leaves

**Figure 5:**
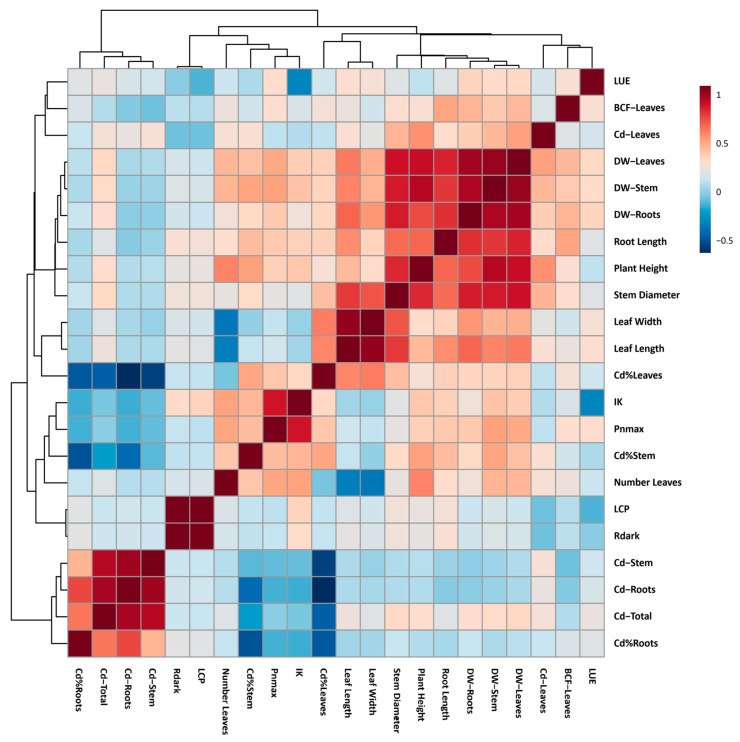
Heat map of Pearson correlation analysis performed in growth, photosynthetic, and cadmium accumulation and allocation variables of four forest species (*C. pyriformis*, *S. macrophylla*, *C. odorata*, and *T. superba*) exposed to contrasting levels and exposure time of cadmium. Light use efficiency (LUE), bioaccumulation factor in leaves (BCF−Leaves), cadmium content in leaves (Cd−Leaves), dry weight of leaves (DW−Leaves), dry weight of stem (DW−Stem), dry weight of roots (DW−Roots), distance from the base of the stem to the tip of the root (Root Length), distance from the base of the stem to the tip of the first leaf (Plant Height), diameter of the median region of the stem (Stem Diameter), width of the third sheet fully expanded (Leaf Width), length of third fully expanded leaf (Leaf Length), percentage of cadmium allocation in leaves (Cd%Leaves), light saturation threshold (IK), maximum photosynthesis not limited by light (Pnmax), percentage of cadmium allocation to stem (Cd%Stem), number of leaves (Number Leaves), light compensation point (LCP), leaf respiration in the dark (Rdark), cadmium content in the stem (Cd−Stem), cadmium content in the roots (Cd−Roots), total cadmium content per plant (Cd−Total), and percentage of cadmium allocation to roots (Cd%Roots).

In order to better understand the correlation between photosynthetic, growth, and cadmium accumulation variables, a Pearson correlation analysis was performed (Figure 5).

In general, the correlation analysis revealed the existence of at least three well-evidenced clusters. The first was formed by the percentage of cadmium allocation in roots (Cd%Roots), the total cadmium content accumulated in the plant (Cd-Total), the cadmium concentration in roots (Cd-Roots), and the cadmium concentration in stems (Cd-Stem). This first group of variables presented a marked positive correlation. The group was formed by the following variables: percentage of cadmium allocation in stems (Cd%Stem), maximum photosynthesis (Pnmax), light saturation threshold (Ik), and percentage of cadmium allocation in leaves (Cd%Leaves). These variables presented a strong negative correlation among themselves. Finally, the cluster formed by the growth variables stands out, which presented a strong positive correlation among themselves.

## 3. Discussion

While global cacao production led by African countries has presented a recent multifactorial bean supply crisis [33], an excellent opportunity for the growth and development of cacao agribusiness in South American countries such as Colombia is expected. However, the bioaccumulation of cadmium in cacao beans in Latin America and the Caribbean, a problem associated with the high level of Cd contamination in the main cacao areas in these countries, is a relevant problem for the commercialization of this product [34]. This need justifies the approach to the problem of excess cadmium in cacao from several fronts. This study shows that forest species associated with cacao, such as *C. pyriformis*, may have a high tolerance to cadmium exposure under concentrations as high as 17 ppm in a solid substrate, with this tolerance at levels comparable to recognized phytoextractor species such as *S. macrophylla* [22]. Furthermore, this study also revealed that both *C. pyriformis* and *T. superba* have a high concentration and allocation of cadmium to the stem, an ideal characteristic in terms of a phytoextraction strategy based on wood harvesting [29]. On the other hand, *C. odorata*, by far the most common timber tree associated with cacao for the quality of its wood, which has made it endangered [35,36], presents an inferior performance in all the phytoextraction criteria listed above, indicating low potential for use for this purpose.

Field experiments using three successive *Brassica juncea* cultivation cycles managed to reduce the lead concentration in acidic soil (pH 5.5) by approximately 30% in the most superficial layers of the soil (0–15 cm), showing that the bioremediation of excess heavy metals in the soil through phytoextraction strategies is a concrete reality [37]. The success of a phytoextraction strategy depends on many factors beyond the species, including the availability of the heavy metal in the soil, which largely depends on lower pH values. Fortunately, in Colombia, most cacao soils have low pH values [38], which strengthens the potential for using cadmium remediation strategies based on phytoextraction. However, little progress has been made despite the strategic potential of using AFS trees for cadmium phytoextraction [29].

Tree species have several advantages from the point of view of their use in phytoextraction concerning herbaceous species. While the growth rates of tree species are often slower than those of herbaceous species, tree species can sustain growth for several years, managing to extract large amounts of potentially toxic elements from the soil over the long term [39,40]. In the field, considering a three-year experiment in the Czech Republic, the tree species *Salix* sp. and *Populus* sp. were able to remove approximately 95 and 66 g of cadmium per hectare [41]. These authors also indicate the importance of the low pH values of the soils in the study area for the phytoextraction result obtained. Other relevant species in terms of the use of trees for the phytoextraction of heavy metals can be *S. macrophylla* [22], neem-*Azadirachta indica* [42], and *Moringa oleifera* [43]. In the present study, using *S. macrophylla* as a reference for a phytoremediation tree species, there is evidence of a high phytoextraction potential exhibited by *C. pyriformis*.

Hyperaccumulation and hypertolerance are critical attributes for selecting a species for the phytoextraction of heavy metals. However, as the present study shows, these two characteristics are not mutually compatible. BCF values greater than 1.0 are generally attributed to hyperaccumulator plants [21]. However, Agrostis tenuis, a perennial grass species belonging to the *Poaceae* family, for example, can accumulate arsenic to concentrations as high as 3470 g kg^−1^ and present a BCF of much less than one [44]. In the present study, considering only the BCF values, *C. odorata* has a potential behaviour of cadmium hyperaccumulation. This species can accumulate approximately two micrograms of cadmium per plant per day, much higher than *S. macrophylla*, a phytoremediator species [22]. However, with TI values approaching 50%, especially in T2 conditions, the feasibility of phytoextraction for *C. odorota* is questioned, especially in higher conditions of cadmium contamination. Ramirez et al. [45] estimated the aboveground biomass of *C. odorata* plantations in Costa Rica of a 19-year-old tree, which can reach a total biomass of 393.2 kg, of which 5.7 kg are leaves and 2.1 kg are petioles, which, due to its deciduous behaviour, can be recycled to the system [46]. If we consider an average density of 110 trees per hectare in a typical CASF and at a rate of around 10% cadmium allocation to leaf tissue, an input of around 150 mg of superficial Cd could be projected per hectare due to an annual leaf abscission event after 19 years.

Indeed, reincorporating the cadmium fixed in its foliage is somewhat problematic for cacao cultivation because of two aspects: (1) it translocates cadmium from deep soil layers and makes them available for use by cacao plants, and (2) it remains leafless during the driest period of the year, which means that cacao is not protected against abiotic stresses such as extreme temperatures, intense solar radiation, and precipitation [47,48]. Moreover, although *C. odorata* is associated with cacao and coffee crops, its establishment within these crops has been chiefly under natural regeneration. However, its planting in new plantations under agroforestry systems has been limited due to attack by *Hypsipyla grandella* [45]. Thus, these data together allow us to consider *C. odorata* in CAFS soils with unfavourable cadmium contamination.

In contrast to *C. odorata*, *C. Pyriformis* and *T. superba* present lower cadmium allocation to the leaves and significant allocation to the stem, reaching 1/4 of all cadmium accumulated per plant in *T. superba*. Plant stem tissues have very peculiar characteristics that deserve attention when we compare them with tissues such as leaves and roots. Three main components can be distinguished in a typical tree: epidermis, cortex, and vascular tissue. Although our data are insufficient to determine which stem tissue predominantly stores cadmium in *T. superba*, it is commonly assumed that a significant portion of this metal may be associated with vascular tissues, therefore suggesting a transient allocation. However, cortex tissues such as parenchyma cells (young plants), collenchyma cells, and especially sclerenchyma cells can have very rigid cell walls due to lignin deposition and provide rigidity to support the tissue [49]. It is important to note that the cell wall components, such as pectin, hemicellulose, and lignin, have functional groups that can bind heavy metal ions, effectively immobilizing them. Notably, the surface of lignin contains functional groups like hydroxyl and carboxyl, which could bind heavy metals and decrease their bioavailability [50].

Moreover, Cd exposure may enhance the biosynthesis of lignin since it may disturb the oxidative balance and increase POD activity, which leads to increased lignification. Under such conditions, the increment in lignin deposition in cell walls may inhibit elongation and plant growth. In addition, lignin and other molecules may prevent further absorption into the protoplast by mounting a physical barrier and immobilizing Cd in the secondary cell wall, which may also be a defence response [51]. This response is similar to that observed in plants exposed to other phytotoxic elements in the soil, such as NaCl [52] and NH_4_^+^ [53]. In this context, the reductions in growth, especially in root length, presented by several species exposed to cadmium could represent a defence mechanism associated with tissue lignification [51]. This growth restriction could or could not be accompanied by eliminating older leaves, highly concentrated in toxic elements, as an additional strategy for detoxifying the plant and keeping photosynthetic tissues free from toxic effects [29]. Nevertheless, future studies are still needed to determine the specific site of Cd accumulation in the stem tissues of species such as *T. superba* and *C. pyriformis* and the roles of lignin in this phenomenon.

Interestingly, the results presented by *T. superba* in terms of cadmium allocation to stem and TI tissues under contamination conditions with 7.6 and 17.4 mg kg^−1^ were like those given by the species under hydroponics conditions (6 and 12 mg kg^−1^) in a similar period of exposure [29]. However, it is essential to highlight the contrast between the amounts of cadmium accumulated in plants, which were up to ten times higher when evaluated in a hydroponic medium. In the same context, *C. pyriformis*, which also had a high concentration of cadmium in stems under hydroponic conditions, had shown a low total accumulation of cadmium per plant, unlike what was observed in the present study, where *C. pyriformis* was only in the background of *C. odorata* in terms of cadmium accumulation per plant. For *C. pyriformis*, however, the big difference between hydroponic and substrate media observed is in growth, which was more than 3× higher in substrate media, which could explain the differences in cadmium accumulated in this species. This result may reveal some species’ difficulty in acclimatizing to a hydroponic environment, making it difficult to assess their phytoextraction capacity under such conditions. Therefore, the present study confirms the high potential for cadmium allocation to the stem of *T. superba* and clarifies the hyperaccumulator potential of *C. pyriformis*, which can be affected by excess water, as occuring in hydroponic media.

Considering the impact of photosynthesis on plant growth, it is interesting to note that *T. superba* is the species that presents the most significant adverse effects on photosynthesis due to exposure to cadmium. Our results highlight the substantial reduction in stomatal conductance in *T. superba*, which is more significant after 30 days of exposure to Cd and leads to significant differences in maximum photosynthesis. This comportment contrasts with the more tolerant species *C. pyriformis* and *S. macrophylla*, which showed fewer differences in stomatal opening. In fact, among the species evaluated here, *T. superba* has the most stomatal opening, which may indicate a high transpiration flow and better adaptability to environments with excess water. On the other hand, a greater transpiration flow in *T. superba* could be associated with the high presence of Cd in stem tissues of this species, which would be transported indirectly by the xylem sap. A high flux of Cd mobilized by the transpiration current, combined with cadmium entrapment in cell wall elements, would form a plausible hypothesis to explain the greater allocation of Cd in *T. superba* stems. However, it is worth highlighting that in *C. pyriformis*, the stomatal opening values are much lower, and the species is also capable of maintaining large fractions of cadmium in stem tissues, which suggests that more mechanisms may co-influence this phenomenon, probably in the species-specific level.

Therefore, our results point to the prominent role of *C. pyriformis* in terms of its phytoextraction potential, indicated by a high tolerance index, combined with high cadmium accumulation and high allocation in stem tissues. Finally, the potential presented by *T. superba* also stands out, considering contamination levels up to 7 mg kg^−1^, given the high transpiration flow of this species under these conditions and the high potential for cadmium allocation in stem tissues. *C. odorata*, on the other hand, despite being one of the main species associated with cacao in Colombia’s CAFSs, presents several negative factors concerning cadmium. Despite accumulating large proportions and presenting a high TI under conditions of ~7 mg kg^−1^ cadmium, it allocates a relatively high fraction of cadmium to leaf tissues. This response, combined with the annual deciduous habit presented by the species, tends to form a system of cadmium capture at depths associated with its deposition on surfaces due to fallen leaves. More studies are needed to understand the biochemical mechanisms associated with cadmium accumulation in stems of *C. pyriformis* and *T. superba*.

## 4. Materials and Methods

### 4.1. Plant Material and Experimental Conditions

The plants were grown under greenhouse conditions at the Agrosavia “La Suiza” Research Center located in the municipality of Rionegro, department of Santander (7°22′12″ N) (73°10′39″ W), located at 530 m above sea level under natural sunlight at an average temperature of 25 ± 4 °C and an average photosynthetic photon flux density of 1390 ± 534 µmol m^−2^ s^−1^ at noon. Certified seeds of the forest species *Cariniana pyriformis* Miers, *Terminalia superba* Engl. and Diels, *Swietenia macrophylla* King, and *Cedrela odorata* L. from the forest seed producer “El semillero S.A.S” were used. Seeds were germinated and developed for 60 days in sphagnum peat arranged in plastic trays. Seedlings that were 60 days old were transferred to 5 kg plastic containers containing a substrate consisting of soil (first 30 cm), fine river sand, and organic compost (3:1:0.5), at pH 6.1 ± 1.8. This substrate was crushed, sieved, and disinfected using the solarization technique with a black plastic cover. Cadmium chloride (CdCl_2_—SIGMA-ALDRICH, Saint Louis, MO, USA; cadmium chloride—99.99% trace metals basis) was used as the source of cadmium. The supply of Cd (T1 = 7 and T2 = 17 mg·kg^−1^) was carried out before transferring all the plants. The contamination was performed by considering the total weight of the substrate as a basis for determining the final total concentration in the plastic containers. Thus, Cd exposure was carried out for up to 90 days after transferring (DAEs) the plants to the previously contaminated substrate. Physiological growth parameters (plant height, basal diameter, and the number of leaves or leaflets) and photosynthesis activity were recorded at 30, 60, and 90 DAEs. Three destructive samplings were also carried out at 30, 60, and 90 DAEs to evaluate the leaf, stem, and root dry mass and cadmium content.

### 4.2. Cadmium Content and Cadmium Tolerance Index

Determination of cadmium was performed by Inductively Coupled Plasma Mass Spectrometry using a mixture comprising 5 mL of HNO_3_ (65%—MERCK, Darmstadt, Germany) and 2 mL of H_2_O_2_ (30%—MERCK) [54]. The methodology proposed by Wilkins [55] was followed to calculate the tolerance index [TI = Total dry weight in Cd exposed plants/Total dry weight in reference plants × 100]. The TI was estimated at each destructive sampling (30, 60, and 90 days after exposure), and an average for the entire period was calculated for each forest species under each Cd exposure level.

For soil, four methods (0.05 M EDTA, 1 M NH_4_OAc, 0.01M CaCl_2_, and Mehlich 3) were compared to identify the best extraction method for predicting available Cd in soils. In brief, 1 g of soil was equilibrated with the soil/reagent ratio of 1:10 for an equilibration time of 2 h and 30 min for NH_4_OAc (PANREAC, Barcelona, Spain) [56]; 2 g of soils was equilibrated with soil/reagent ratio of 1:20 for an equilibration time of 1 h for CaCl_2_ (MERCK); 2 g of soils was equilibrated with soil/reagent ratio of 1:20 for an equilibration time of 10 min for EDTA (SIGMA-ALDRICH) [57]; and finally, 3 g of soils was equilibrated with soil/reagent ratio of 1:10 for an equilibration time of 5 min for Mehlich 3 (0.2 M CH_3_COOH (MERCK) + 0.25 M NH_4_NO_3_ (SIGMA-ALDRICH) + 0.015 M NH_4_F (MERCK) + 0.013 HNO_3_ (65%—MERCK) + 0.001 M EDTA (SIGMA-ALDRICH), pH 2) [58]. Also, it was realized that the total recovered Cd was estimated using acid digestion with nitric (65%—MERCK) and perchloric acid (70–72%—MERCK) (3:1 *v*/*v*).

### 4.3. Photosynthesis

A portable infrared gas analyser system (LI-6800, LICOR, Lincoln, NE, USA) was used to measure gas exchange parameters in forest species. This analysis employed mature leaves (generally 3rd from the top) previously acclimated to growth light conditions. The variables of net CO_2_ assimilation (A), total transpiration rate (E), and stomatal conductance to water (gSw) were determined. Leaf water use efficiency (WUEl) was also estimated as A/E. The temperature inside the measurement chamber was kept at 28 °C, and the CO_2_ partial pressure was set to 400 ppm. In all measurements, the amount of blue light was set up to 10% of the PPFD to maximize stomatal aperture [59], and the leaf-to-air vapour pressure difference was 1.85 ± 0.14 kPa. Light curves were obtained by changing PPFD from 2000 μmol m^−2^ s^−1^ to 0, where measurements were recorded when the total coefficient of variation was lower than 5%, and temporal stability was achieved (on average, 3 min after the beginning of each test). All the determinations were performed between 8:00 am and 2:00 pm.

### 4.4. Experimental Design and Statistical Analysis

The experiment was arranged under a completely randomized factorial design (3 × 4, three cadmium levels, and four forest species), where 18 plants for each treatment were used as experimental units, with three repetitions for each experimental unit. From this experimental unit, six plants were taken to assess cadmium content and destructive sampling at 30, 60, and 90 DAEs. A two-way ANOVA (*p* ≤ 0.05) was performed for each parameter evaluated. Tukey’s test (*p* < 0.05) was performed when significant differences were detected to evaluate contrasts between cadmium treatments and plant species. The obtained data were used to perform a Pearson correlation analysis. First, the data were normalized using log base 10 and Pareto scaling. Then, the correlation data were used to construct a heat map, using MetaboAnalystR: https://github.com/xia-lab/MetaboAnalystR (accessed on 1 February 2025).

## Figures and Tables

**Table 1:** Tolerance index in four forest species (*C. pyriformis*, *S. macrophilla*, *C. oderata*, and *T. superba*) exposed to contrasting levels of cadmium (T1 and T2) at 30, 60, and 90 days after cadmium exposure (DAEs). Mean values ± standard error (*n* = 3) is shown. Different capital letters represent significant differences (Tukey, *p* ≤ 0.05) between plant species under the same Cd treatment and time of exposure. Different lowercase letters mean significant differences (Tukey, *p* ≤ 0.05) between cadmium treatments within the same plant species and time of exposure.

Species	Cadmium	Tolerance Index (%)		
		30 DAEs	60 DAEs	90 DAEs
*C. pyriformis*	T1	66 ± 8 Aa	96 ± 7 Aa	100 ± 7 Aa
	T2	67 ± 6 Aa	68 ± 21 Aa	84 ± 5 Aa
*S. macrophylla*	T1	83 ± 6 Aa	84 ± 3 Aa	97 ± 16 Aa
	T2	86 ± 13 Aa	73 ± 5 Aa	98 ± 8 Aa
*C. odorata*	T1	82 ± 15 Aa	72 ± 3 Aa	91 ± 17 Aa
	T2	65 ± 5 Aa	64 ± 7 Aa	54 ± 13 Ba
*T. superba*	T1	78 ± 14 Aa	68 ± 7 Aa	80 ± 5 Aa
	T2	63 ± 9 Aa	53 ± 5 Ba	52 ± 12 Bb

**Table 2:** Cadmium content (mg kg^−1^) in four young forest tree species related to cacao agroforestry systems (*C. pyriformis*, *S. macrophilla*, *C. oderata*, and *T. superba*) exposed to different levels of CdCl_2_ contamination (reference, T1, and T2) at 30, 60, and 90 days. The total average (AVR, *n* = 3) and standard error of the mean (SEM) are shown. Different capital letters represent significant differences (Tukey, *p* ≤ 0.05) between plant species under the same Cd treatment and time of exposure. Different lowercase letters mean significant differences (Tukey, *p* ≤ 0.05) between cadmium treatments within the same plant species and time of exposure.

	Time (DAEs)	Cd	*C. pyriformis*	*S. macrophilla*	*C. odorata*	*T. superba*
Leaf Cadmium Content (mg kg^−1^)	30	Reference	0.03 ± 0.00 aA	0.40 ± 0.2 aA	0.25 ± 0.01 bA	0.43 ± 0.18 aA
		T1	0.52 ± 0.11 aA	2.01 ± 0.12 aA	5.11 ± 0.35 aA	1.96 ± 0.35 aA
		T2	1.96 ± 0.86 aB	3.96 ± 0.44 aA	8.92 ± 1.21 aA	3.62 ± 0.18 aA
	60	Reference	0.04 ± 0.00 aA	0.36 ± 0.18 aA	0.21 ± 0.02 cA	0.25 ± 0.02 aA
		T1	0.82 ± 0.13 aB	2.95 ± 1.12 aA	7.39 ± 0.39 bA	2.34 ± 0.02 aA
		T2	2.28 ± 0.08 aB	4.16 ± 0.45 aB	15.24 ± 3.5 aA	4.04 ± 0.48 aB
	90	Reference	0.04 ± 0 aA	0.13 ± 0.03 bA	0.29 ± 0.04 cA	0.28 ± 0.02 bA
		T1	1.43 ± 0.26 aB	2.66 ± 0.21 aB	12.61 ± 0.85 bA	5.04 ± 0.22 bB
		T2	5.53 ± 1.48 aC	6.74 ± 2.03 aC	35.35 ± 1.14 aA	13.34 ± 3.66 aB
Stem Cadmium Content (mg kg^−1^)	30	Reference	0.07 ± 0.01 cA	0.25 ± 0.03 cA	0.18 ± 0.01 bA	0.22 ± 0.04 bA
		T1	28.53 ± 19.19 aA	6.5 ± 0.36 bB	3.1 ± 0.26 bB	4.53 ± 0.94 bB
		T2	18.04 ± 2.35 cA	17.39 ± 1.7 aA	10.66 ± 3.77 aC	13.96 ± 1.81 aA
	60	Reference	0.67 ± 0.35 cA	0.16 ± 0.01 cA	0.24 ± 0.05 cA	0.23 ± 0.01 cA
		T1	16.17 ± 3.97 bA	9.09 ± 1.51 bB	6.6 ± 0.46 bB	12.42 ± 2.64 bA
		T2	37.16 ± 4.39 aA	16.81 ± 3.28 aC	14.37 ± 4.29 aC	24.11 ± 1.29 aB
	90	Reference	0.06 ± 0.01 cA	0.31 ± 0.08 cA	0.31 ± 0.03 cA	0.41 ± 0.06 cA
		T1	21.49 ± 1.27 bA	13.93 ± 0.67 bD	20.05 ± 4.78 bB	26.26 ± 5.71 bA
		T2	93.66 ± 12.36 aA	28.84 ± 1.84 aD	44.9 ± 7.87 aC	78.77 ± 2.57 aB
Roots Cadmium Content (mg kg^−1^**)**	30	Reference	0.45 ± 0.07 cA	0.51 ± 0.07 cA	0.27 ± 0.04 cA	0.71 ± 0.39 cA
		T1	82.08 ± 31.12 bC	65.44 ± 3.9 bD	135.3 ± 11.99 bA	100.61 ± 14.57 bB
		T2	357.95 ± 15.4 aA	153.57 ± 2.73 aD	280.72 ± 82.35 aC	294.61 ± 17.06 aB
	60	Reference	0.60 ± 0.07 cA	0.39 ± 0.03 cA	0.5 ± 0.03 cA	0.51 ± 0.09 cA
		T1	283.77 ± 52.14 bB	74.77 ± 8.2 bD	311.62 ± 17.25 bA	156.53 ± 8.85 bC
		T2	776.19 ± 145.91 aA	149.39 ± 20.21 aD	666.52 ± 254.28 aB	384.58 ± 162.92 aC
	90	Reference	3.01 ± 2.05 cA	1.78 ± 0.25 cA	1.15 ± 0.27 cA	1.01 ± 0.14 cA
		T1	240.23 ± 34.91 bC	156.61 ± 4.11 bD	337.93 ± 65.01 bB	440.34 ± 99.8 bA
		T2	652.99 ± 18.31 aA	295.13 ± 42.14 aD	613.35 ± 71.18 aC	635.39 ± 121.37 aB

## Data Availability

Data are contained within the article and Supplementary Materials.

## Supplementary Materials

The following supporting information can be downloaded at https://www.mdpi.com/article/10.3390/plants14071101/s1. Figure S1: Cadmium content for contrasting contamination levels (Reference, T1, and T2) after 90 days of exposure. The bars represent mean values ± standard error (n = 3). According to Tukey’s test (*p* < 0.05), different letters mean significant differences between cadmium treatments; Figure S2: Light curves in four forest species (*C. pyriformis*, *S. macrophylla*, *C. odorata*, and *T. superba*) exposed to contrasting levels of cadmium contamination (T1 and T2) for 30 days. Photosynthetic light curves, A-PPFD (left panel). Stomatal conductance light curves, gSw-PPFD (centre panel) and water use efficiency response to light curves, WUE-PPFD (right panel) in response to differences in photosynthetic photons flux density (PPFD). Circles represent mean values ± standard error (n = 3). A-PPFD curves were modelled by non-linear regression according to Lieth and Reynolds 1987; Figure S3: Light curves in four forest species (*C. pyriformis*, *S. macrophylla*, *C. odorata*, and *T. superba*) exposed to contrasting levels of cadmium contamination (T1 and T2) for 60 days. Photosynthetic light curves, A-PPFD (left panel). Stomatal conductance light curves, gSw-PPFD (centre panel) and water use efficiency response to light curves, WUE-PPFD (right panel) in response to differences in photosynthetic photons flux density (PPFD). Circles represent mean values ± standard error (n = 3). A-PPFD curves were modelled by non-linear regression according to Lieth and Reynolds 1987; Figure S4: Light curves in four forest species (*C. pyriformis*, *S. macrophylla*, *C. odorata*, and *T. superba*) exposed to contrasting levels of cadmium contamination (T1 and T2) for 90 days. Photosynthetic light curves, A-PPFD (left panel); Stomatal conductance light curves, gSw-PPFD (centre panel) and water use efficiency response to light curves, WUE-PPFD (right panel) in response to differences in photosynthetic photons flux density (PPFD). Circles represent mean values ± standard error (n = 3). A-PPFD curves were modelled by non-linear regression according to Lieth and Reynolds 1987; Table S1: Growth variables in four young forest tree species related to cacao agroforestry systems (*C. pyriformis*, *S. macrophylla*, *C. odorata*, and *T. superba*) exposed to different levels of CdCl_2_ contamination (reference, T1, and T2) at 30, 60, and 90 days after exposure. The total average (n = 3) and standard error of the mean (SEM) are shown. Different capital letters represent significant differences (Tukey, *p* ≤ 0.05) between plant species under the same Cd treatment and time of exposure. Different minor letters mean significant differences (Tukey, *p* ≤ 0.05) between cadmium treatments within the same plant species and exposure time; Table S2: Relative growth rate (RGR) in four young forest tree species related to cacao agroforestry systems and exposed to CdCl_2_ (reference, T1, and T2) for up to 90 days. The total average (n = 3) and standard error of the mean (SEM) are shown; Table S3: Cadmium bioconcentration factor (BCF) in leaves of four young forest tree species related to cacao agroforestry systems and exposed to CdCl_2_ (T1 and T2) for up to 90 days. The total average (n = 3) and standard error of the mean (SEM) are shown. Different capital letters represent significant differences (Tukey, *p* ≤ 0.05) between plant species under the same Cd treatment and time of exposure. Different minor letters mean significant differences (Tukey, *p* ≤ 0.05) between cadmium treatments within the same plant species and exposure time; Table S4: Light requirements based on A-PPFD fitted curves of four young forest tree species related to cacao agroforestry systems and exposed to CdCl_2_ (reference, T1 and T2) for up to 90 days. The onset of light saturation (IK), the light required for 90% of maximum assimilation (L90), and the light compensation point (LCP) are shown. The total average (n = 3) and standard error of the mean (SEM) are shown. Different capital letters represent significant differences (Tukey, *p* ≤ 0.05) between plant species under the same Cd treatment and time of exposure. Different minor letters mean significant differences (Tukey, *p* ≤ 0.05) between cadmium treatments within the same plant species and exposure time. The total average (n = 3) and standard error of the mean (SEM) are shown.

## Abbreviations

The following abbreviations are used in this manuscript:

AFSs	Agroforestry systems
CAFSs	Agroforestry systems with cacao
PPFD	Photosynthetic photons flux density
Amax	Maximum CO_2_ assimilation not limited by light
LUE	Maximum photosynthetic light use efficiency
Rdark	Mitochondrial respiration in the dark
gSw	Stomatal conductance
WUEl	Leaf level water use efficiency
