# Supplementary material for: Phytoextraction and Cd Allocation to the Stem of Woody Species Used in Cacao Agroforestry
Source: Plants (Basel). 2025 Apr 2;14(7):1101. doi: 10.3390/plants14071101 (PMC11991088; doi:10.3390/plants14071101)
Supplement: Supplementary file 1 [file plants-14-01101-s001.zip › plants-3538865-supplementary.pdf]

Phytoextraction and Cd allocation to the stem of woody species used in cacao agroforestry

Fabricio E. L. Carvalho[a*]; Andrea C. Montenegro[b]; Laura D. Escobar-Pachajoa[a]; Jairo Rojas-Molina[a]; Jorge E. Camacho-Diaz[a]; Gersain A. Rengifo-Estrada[a]

[a]Centro de Investigación La Suiza, Corporación Colombiana de Investigación Agropecuaria - AGROSAVIA, Santander, Colombia.

[b]Centro de Investigación Tibaitatá, Corporación Colombiana de Investigación Agropecuaria - AGROSAVIA, Cundinamarca, Colombia.

Supplementary Material

2025

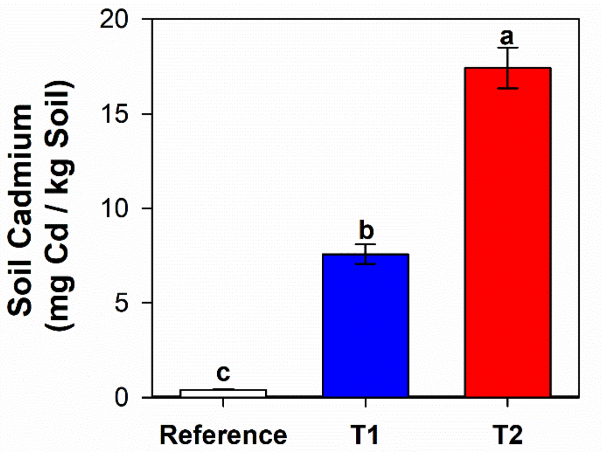

Figure S1. Cadmium content for contrasting levels of contamination (Reference, T1 and T2) after 90 days of exposure. The bars represent mean values ± standard error ($n = 3$). Different letters mean significant differences between cadmium treatments, according to Tukey's test ($p < 0.05$).

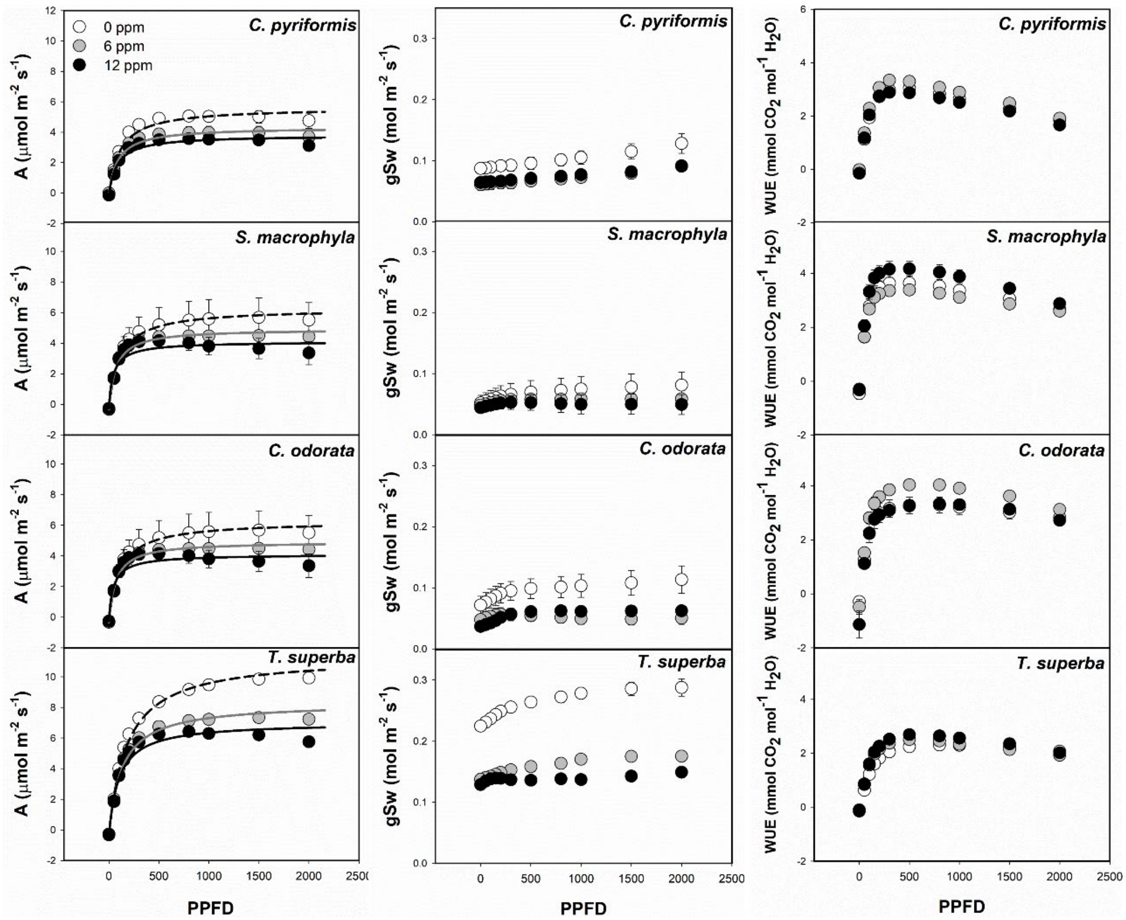

Figure S2. Light curves in four forest species (*C. pyriformis*, *S. macrophylla*, *C. oderata*, and *T. superba*) exposed to contrasting levels of cadmium contamination (T1 and T2) for 30 days. Photosynthetic light curves, A-PPFD (left panel); Stomatal conductance light curves, gSw-PPFD (center panel) and water use efficiency light curves, WUE-PPFD (right panel) in response to differences in photosynthetic photons flux density (PPFD). Circles represent mean values ± standard error (n = 3). A-PPFD curves were modeled by non-linear regression according to Lieth and Reynolds 1987.

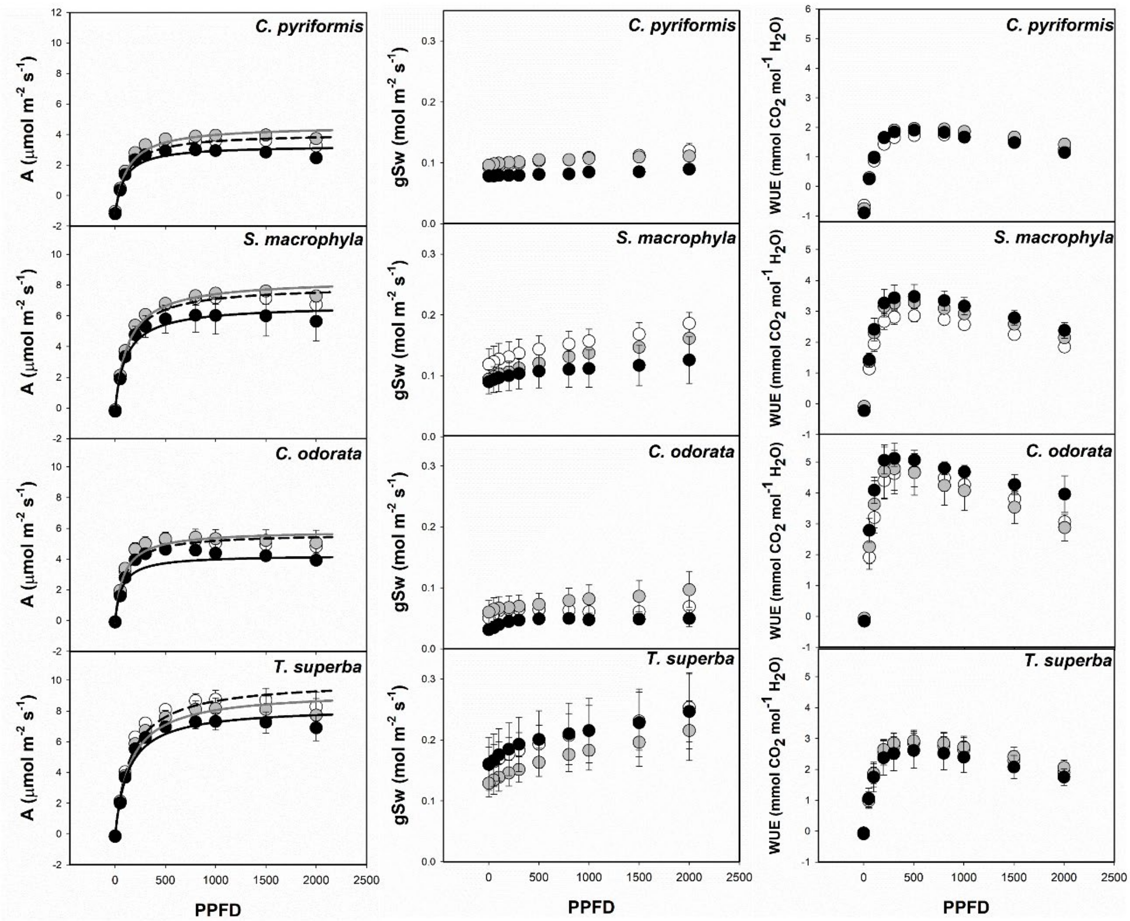

Figure S3. Light curves in four forest species (*C. pyriformis*, *S. macrophylla*, *C. oderata*, and *T. superba*) exposed to contrasting levels of cadmium contamination (T1 and T2) for 60 days. Photosynthetic light curves, A-PPFD (left panel); Stomatal conductance light curves, gSw-PPFD (center panel) and water use efficiency light curves, WUE-PPFD (right panel) in response to differences in photosynthetic photons flux density (PPFD). Circles represent mean values ± standard error (n = 3). A-PPFD curves were modeled by non-linear regression according to Lieth and Reynolds 1987.

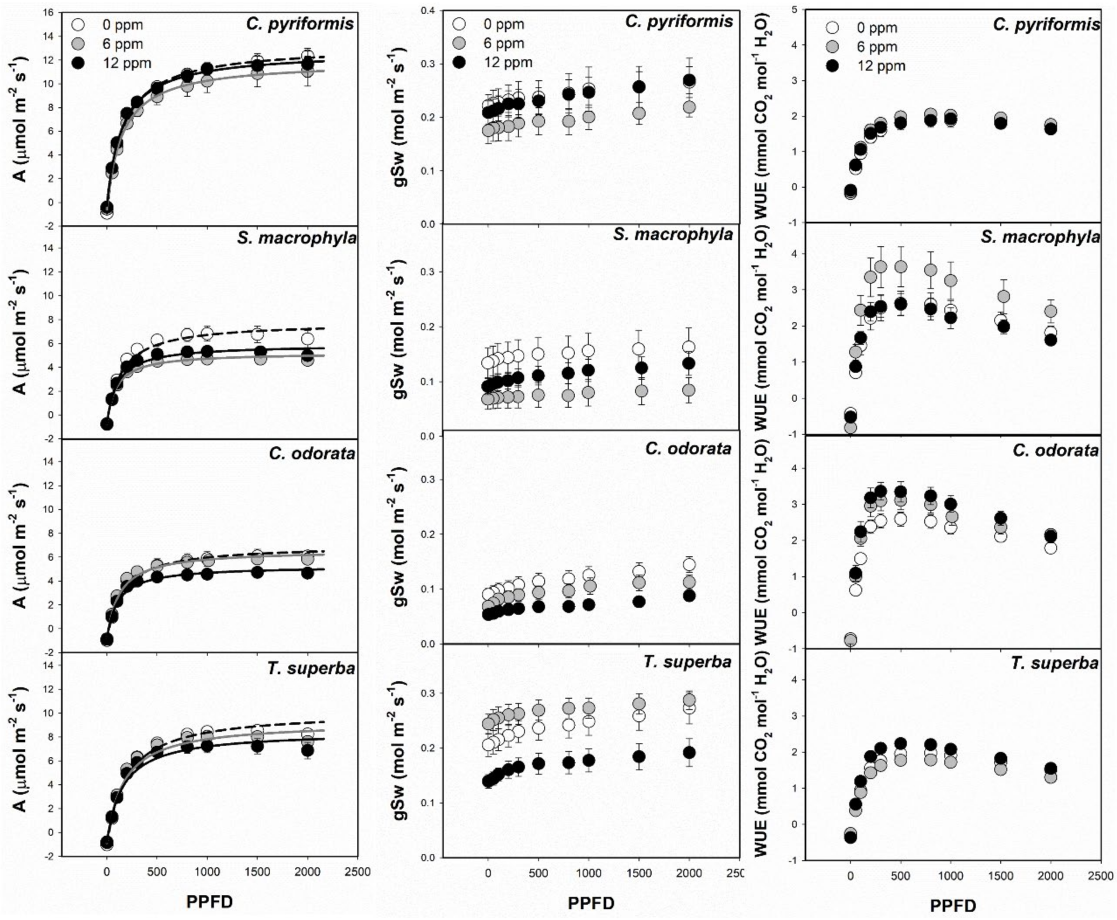

Figure S4. Light curves in four forest species (*C. pyriformis*, *S. macrophylla*, *C. oderata*, and *T. superba*) exposed to contrasting levels of cadmium contamination (T1 and T2) for 90 days. Photosynthetic light curves, A-PPFD (left panel); Stomatal conductance light curves, gSw-PPFD (center panel) and water use efficiency light curves, WUE-PPFD (right panel) in response to differences in photosynthetic photons flux density (PPFD). Circles represent mean values ± standard error (n = 3). A-PPFD curves were modeled by non-linear regression according to Lieth and Reynolds 1987.

Table S1. Growth variables in four young forest tree species related to cocoa agroforestry systems (*C. pyriformis*, *S. macrophylla*, *C. oderata*, and *T. superba*) exposed to different levels of $CdCl_2$ contamination (reference, T1 and T2) at 30, 60 and 90 days after exposure. The total average (n=3) and standard error of the mean (SEM) are shown. Different capital letters represent significant differences (Tukey, p≤0.05) between plant species under the same Cd treatment and time of exposure. Different minor letters mean significant differences (Tukey, p≤0.05) between cadmium treatments within the same plant species and time of exposure.

Time (days)	Species	Cd	Stem diameter (mm)	SEM (±)	Tukey	Leaves (number)	SEM (±)	Tukey	Leaf area (cm^2)	SEM (±)	Tukey
30	***C. odorata***	Reference	2,8	0,1	*Aa*	7,3	0,3	*Ba*	164,2	16,7	*Aa*
		T1	2,4	0,1	*Aa*	6,9	0,4	*Ba*	148,8	13,5	*Aab*
		T2	2,3	0,2	*Ba*	6,2	0,6	*Ba*	113,2	12,4	*Ab*
	C. pyriformis	Reference	2,0	0,1	*Ba*	14,1	1,2	*Aa*	10,5	2,2	*Ba*
		T1	1,9	0,1	*Ba*	11,1	0,7	*Aab*	8,1	0,3	*Ca*
		T2	1,8	0,1	*Ca*	8,3	0,3	*Ab*	7,7	0,4	*Ba*
	S. macrophyla	Reference	2,6	0,1	*Aa*	6,4	0,2	*Ba*	106,9	9,5	*Aa*
		T1	2,5	0,1	*Aa*	6,2	0,2	*Ba*	84,8	7,3	*Aba*
		T2	2,8	0,1	*Aa*	6,2	0,2	*Ba*	80,4	9,4	*Aa*
	T. superba	Reference	1,9	0,1	*Ba*	20,9	7,5	*Aa*	48,2	2,9	*Ba*
		T1	1,8	0,1	*Ba*	14,4	5,5	*Aab*	36,3	1,5	*Bb*
		T2	1,4	0,1	*Db*	8,5	0,2	*Ab*	33,3	1,4	*Bb*
60	***C. odorata***	Reference	4,5	0,3	*Aa*	10,9	0,4	*Ba*	337,1	31,0	*Aa*
		T1	3,9	0,3	*Aa*	10,8	0,2	*Ba*	292,9	21,6	*Aa*
		T2	3,7	0,2	*Aa*	10,6	0,4	*Ba*	261,8	18,9	*Aa*
	C. pyriformis	Reference	2,5	0,1	*Ba*	33,0	1,4	*Aa*	10,0	0,4	*Ca*
		T1	2,4	0,1	*Ba*	25,1	2,3	*Aab*	9,3	0,4	*Ca*
		T2	2,4	0,1	*BCa*	19,1	1,8	*Ab*	11,0	1,0	*Ca*
	S. macrophyla	Reference	2,9	0,1	*Ba*	8,7	0,3	*Ca*	117,1	7,6	*Ba*
		T1	2,9	0,1	*ABa*	8,7	0,3	*Ca*	95,8	8,6	*Ba*
		T2	2,8	0,1	*Ba*	7,9	0,4	*Ca*	90,6	6,3	*Ba*
	T. superba	Reference	2,8	0,2	*Ba*	14,4	0,4	*ABa*	82,4	5,3	*BCa*
		T1	2,4	0,2	*Bab*	13,5	0,3	*ABa*	71,3	5,9	*Ba*
		T2	2,1	0,1	*Cb*	12,1	0,7	*ABa*	59,5	5,7	*Ba*
90	***C. odorata***	Reference	6,4	0,4	*Aa*	13,3	0,6	*Ba*	442,4	32,3	*Aa*
		T1	6,0	0,3	*Aa*	14,3	0,6	*BCa*	400,9	20,9	*Aa*
		T2	5,0	0,4	*Aa*	11,6	0,9	*Ba*	397,3	45,5	*Aa*
	C. pyriformis	Reference	3,1	0,1	*Cab*	53,2	3,3	*Aa*	17,1	4,7	*Ca*
		T1	3,2	0,1	*Ba*	52,4	3,4	*Aa*	12,4	0,9	*Cab*
		T2	3,0	0,1	*Bb*	47,5	1,6	*Aa*	9,8	0,4	*Cb*
	S. macrophyla	Reference	4,0	0,2	*Ba*	11,3	0,8	*Ca*	148,3	19,6	*Ba*
		T1	3,6	0,1	*Ba*	10,7	0,4	*Ca*	132,8	5,9	*Aba*
		T2	3,7	0,3	*ABa*	10,0	0,3	*Ba*	205,7	52,3	*Aba*
	T. superba	Reference	4,4	0,3	*ABa*	17,8	0,6	*ABa*	111,3	6,2	*Ba*
		T1	3,5	0,3	*Bab*	17,3	0,7	*Ba*	95,8	8,5	*Ba*
		T2	3,2	0,2	*Bb*	15,9	0,9	*Ba*	89,7	7,8	*Ba*

Table S2. Relative Growth Rate (RGR) in four young forest tree species related to cocoa agroforestry systems and exposed to $CdCl_2$ (reference, T1 and T2) for up to 90 days. The total average (n=3) and standard error of the mean (SEM) are shown.

Cadmium	Species	30-60 DAS			60-90 DAS		
		RGR		SEM	RGR		SEM
Reference	*C. pyrriformis*	0.0083	±	0.0024	0.0430	±	0.0027
	S. macrophylla	0.0279	±	0.0022	0.0037	±	0.0030
	C. odorata	0.0552	±	0.0008	0.0209	±	0.0033
	T. superba	0.0466	±	0.0026	0.0181	±	0.0042
T1	*C. pyrriformis*	0.0214	±	0.0059	0.0446	±	0.0019
	S. macrophylla	0.0284	±	0.0008	0.0073	±	0.0037
	C. odorata	0.0525	±	0.0066	0.0270	±	0.0039
	T. superba	0.0428	±	0.0073	0.0240	±	0.0063
T2	*C. pyrriformis*	0.0014	±	0.0165	0.0575	±	0.0141
	S. macrophylla	0.0233	±	0.0025	0.0135	±	0.0023
	C. odorata	0.0540	±	0.0053	0.0119	±	0.0039
	T. superba	0.0414	±	0.0066	0.0152	±	0.0056

Table S3. Cadmium bioconcentration factor (BCF) in leaves of four young forest tree species related to cocoa agroforestry systems and exposed to $CdCl_2$ (T1 and T2) for up to 90 days. The total average (n=3) and standard error of the mean (SEM) are shown. Different capital letters represent significant differences (Tukey, $p \leq 0.05$) between plant species under the same Cd treatment and time of exposure. Different minor letters mean significant differences (Tukey, $p \leq 0.05$) between cadmium treatments within the same plant species and time of exposure.

Cd	Time (days)	*C. pyriformis*	SEM (±)	Tukey	*S. macrophyla*	SEM (±)	Tukey	*C. odorata*	SEM (±)	Tukey	*T. superba*	SEM (±)	Tukey
	30	0.100	0.015	*aC*	0.197	0.013	*aB*	0.619	0.043	*aA*	0.219	0.034	*aB*
T1	60	0.095	0.015	*aC*	0.358	0.128	*aC*	1.633	0.100	*aA*	0.288	0.006	*aB*
	90	0.311	0.066	*aC*	0.381	0.032	*aC*	1.491	0.078	*aA*	0.569	0.028	*aB*
	30	0.121	0.049	*aC*	0.195	0.023	*aBC*	0.400	0.047	*bA*	0.232	0.021	*aB*
T2	60	0.140	0.007	*aC*	0.277	0.027	*aB*	0.815	0.194	*bA*	0.260	0.032	*aB*
	90	0.553	0.135	*aBC*	0.354	0.109	*aC*	1.435	0.091	*aA*	0.871	0.203	*aB*

Table S4. Correlations between Cd content in the soil and the content in different plant organs.

**Plant organ**	**30 DAS**	**60 DAS**	**90 DAS**
		**r**	
Leaves	0.71ns	0,63 ns	0.89 ns
Stem	0.93 ns	0.91*	0.71ns
Roots	0.89 *	0.86 *	0.91*

* = significant at p≤0.05; ns = not significant at p≤0.05 (Pearson correlation)

Table S5. Light requirements based on A-PPFD fitted curves of four young forest tree species related to cocoa agroforestry systems and exposed to $CdCl_2$ (reference, T1 and T2) for up to 90 days. The onset of light saturation (IK), the light required for 90% of maximum assimilation (L90) and the light compensation point (LCP) are shown. The total average (n=3) and standard error of the mean (SEM) are shown. Different capital letters represent significant differences (Tukey, $p \leq 0.05$) between plant species under the same Cd treatment and time of exposure. Different minor letters mean significant differences (Tukey, $p \leq 0.05$) between cadmium treatments within the same plant species and time of exposure. The total average (n=3) and standard error of the mean (SEM) are shown.

Time (days)	Species	Cd	IK	SEM (±)	Tukey	L90	SEM (±)	Tukey	LCP	SEM (±)	Tukey
30	*C. odorata*	Reference	130.9	10.8	Aa	1242.0	103.0	Aa	6.2	0.5	Aa
		T1	79.9	17.8	Aa	780.3	167.5	ABa	5.5	2.5	Aa
		T2	120.5	22.6	Aa	1264.3	261.2	Aa	15.4	5.4	Aa
	C. pyriformis	Reference	108.5	16.4	Aa	1005.7	156.1	Aa	2.8	1.2	Aa
		T1	91.0	6.6	Aa	825.7	60.2	ABa	0.7	0.5	Ba
		T2	83.4	14.1	Aa	778.3	119.2	Aa	2.7	0.9	Aa
	S. macrophylla	Reference	97.9	22.6	Aa	932.7	187.4	Aa	4.7	1.3	Aa
		T1	68.3	1.5	Aa	660.5	1.6	Ba	4.3	1.1	Aa
		T2	44.8	16.5	Aa	433.0	162.5	Aa	2.9	1.3	Aa
	T. superba	Reference	178.9	5.6	Aa	1661.0	48.8	Aa	5.1	0.2	Aa
		T1	131.4	9.1	Aab	1225.5	82.7	Aab	4.2	0.1	Aa
		T2	100.4	5.3	Ab	951.0	61.3	Ab	4.5	1.3	Aa
60	*C. odorata*	Reference	89.3	11.6	Aa	827.7	111.2	Aa	2.3	1.7	Ba
		T1	81.2	17.2	Aa	745.0	162.3	Aa	1.4	0.8	Ba
		T2	78.8	1.1	Aa	725.0	12.8	Aa	1.6	0.8	Ba
	C. pyriformis	Reference	113.3	1.1	Aa	1323.5	42.1	Aa	23.7	3.2	Aa
		T1	116.6	8.1	Aa	1364.0	94.8	Aa	24.8	1.7	Aa
		T2	87.4	2.1	Aa	1131.0	31.8	Aa	24.7	0.8	Aa
	S. macrophylla	Reference	118.8	4.5	Aa	1093.7	23.6	Aa	2.3	1.6	Ba
		T1	128.4	8.3	Aa	1174.3	73.6	Aa	1.9	0.1	Ba
		T2	98.4	26.1	Aa	912.3	229.2	Aa	2.6	0.7	Ba
	T. superba	Reference	151.9	17.9	Aa	1387.3	166.9	Aa	2.1	1.2	Ba
		T1	146.5	20.0	Aa	1341.0	181.7	Aa	2.2	0.7	Ba
		T2	127.6	20.1	Aa	1173.3	183.4	Aa	2.5	0.3	Ba
90	*C. odorata*	Reference	145.4	8.0	Aa	1524.0	54.3	Aa	18.6	1.3	Aa
		T1	109.1	15.7	ABa	1139.0	148.1	ABa	13.7	0.9	Aa
		T2	102.9	10.5	Aa	1108.7	124.0	Aa	15.5	2.4	Aa
	C. pyriformis	Reference	154.9	13.8	Aa	1500.3	131.3	Aa	9.9	2.5	Ba
		T1	148.2	12.2	ABa	1406.7	126.4	ABa	6.8	3.0	Aa
		T2	151.5	17.1	Aa	1404.3	158.3	Aa	4.0	1.1	Aa
	S. macrophylla	Reference	138.4	16.1	Aa	1387.7	150.6	Aa	12.9	0.7	ABa
		T1	89.8	8.6	Ba	940.7	74.6	Ba	11.5	0.2	Aa
		T2	101.8	5.8	Aa	1044.7	47.3	Aa	11.4	0.3	Aa
	T. superba	Reference	189.8	8.9	Aa	1906.0	80.1	Aa	18.0	0.2	Aa
		T1	167.3	3.9	Aa	1665.3	38.1	Aa	14.6	0.3	Ab
		T2	156.8	12.7	Aa	1563.7	111.9	Aa	13.9	0.7	Ab
